# The Extracellular Loop 2 (ECL2) of the Human Histamine H_4_ Receptor Substantially Contributes to Ligand Binding and Constitutive Activity

**DOI:** 10.1371/journal.pone.0117185

**Published:** 2015-01-28

**Authors:** David Wifling, Günther Bernhardt, Stefan Dove, Armin Buschauer

**Affiliations:** Institute of Pharmacy, Pharmaceutical/Medicinal Chemistry II, University of Regensburg, Regensburg, Germany; University of South Florida College of Medicine, UNITED STATES

## Abstract

In contrast to the corresponding mouse and rat orthologs, the human histamine H_4_ receptor (hH_4_R) shows extraordinarily high constitutive activity. In the extracellular loop (ECL), replacement of F169 by V as in the mouse H_4_R significantly reduced constitutive activity. Stabilization of the inactive state was even more pronounced for a double mutant, in which, in addition to F169V, S179 in the ligand binding site was replaced by M. To study the role of the FF motif in ECL2, we generated the hH_4_R-F168A mutant. The receptor was co-expressed in Sf9 insect cells with the G-protein subunits Gα_i2_ and Gβ_1_γ_2_, and the membranes were studied in [^3^H]histamine binding and functional [^35^S]GTPγS assays. The potency of various ligands at the hH_4_R-F168A mutant decreased compared to the wild-type hH_4_R, for example by 30- and more than 100-fold in case of the H_4_R agonist UR-PI376 and histamine, respectively. The high constitutive activity of the hH_4_R was completely lost in the hH_4_R-F168A mutant, as reflected by neutral antagonism of thioperamide, a full inverse agonist at the wild-type hH_4_R. By analogy, JNJ7777120 was a partial inverse agonist at the hH_4_R, but a partial agonist at the hH_4_R-F168A mutant, again demonstrating the decrease in constitutive activity due to F168A mutation. Thus, F168 was proven to play a key role not only in ligand binding and potency, but also in the high constitutive activity of the hH_4_R.

## Introduction

**Figure 1:**
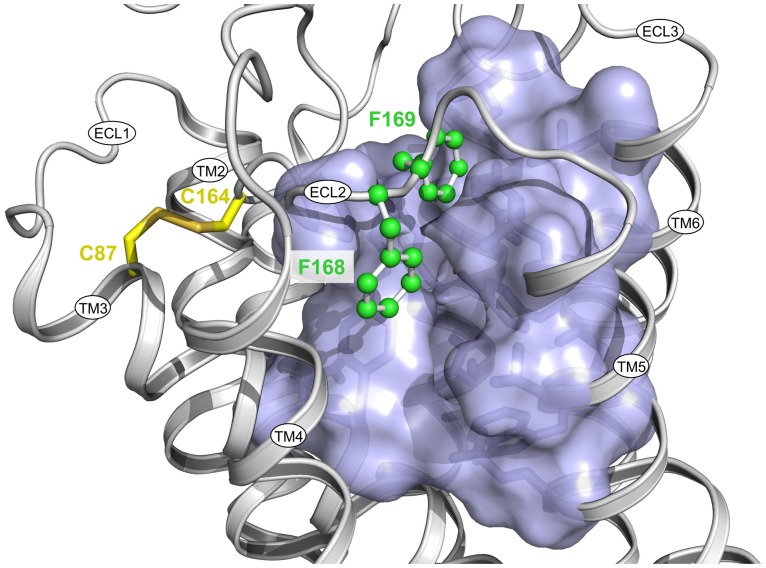
View from the extracellular side into the binding pocket of the human H_4_R. Homology model [9] based on the crystal structure of the hH_1_R inactive state [24]. The FF motif (F168 and F169), pointing to the ligand binding pocket, is illustrated as green balls and sticks, the disulfide bond connecting TM3 with ECL2 as yellow sticks and the binding pocket as a semitransparent surface colored in magenta. Generated with PyMOL Molecular Graphics System, Version 1.6 (Schrödinger LLC, Portland, OR, USA).

Among the extracellular loops (ECLs) of class A GPCRs, the ECL2 is the largest and the most diverse one [1]. ECL2 contributes to ligand recognition, binding, selectivity, allosteric modulation and activation of GPCRs [1, 2]. In the absence of ligand, ECL2 is a putative “gatekeeper” [1], assumed to adopt an open conformation giving access to the binding pocket. Ligand binding can induce a partially closed conformation. Massotte et al. [3] and Klco et al. [4] suggested that ECL2 is involved in interactions stabilizing the inactive state of the receptor. However, specific amino acid sequences in the ECL2 of some GPCRs may stabilize active receptor states and play a role in constitutive activity [5, 6]. For instance, ECL2 was reported to be involved in the activation of the human muscarinic M_3_ (hM_3_R) [7] and the human histamine H_4_ receptor (hH_4_R) [8, 9]. Additionally, the disulfide bond between cysteines in both ECL2 and transmembrane domain 3 (TM3, Fig. 1) is of relevance for GPCR function, as shown, for example, for rhodopsin [10], the M_1_R [11], the β_2_-adrenergic (β_2_AR) [12] and the gonadotropin releasing hormone receptor (GnRH-R) [13]. Furthermore, ECL2 contributes to the high affinity state of the β_2_AR [12]. Apart from modifying ligand-free states, ECL2 was shown to have an impact on ligand binding and selectivity [11, 14, 15].

Constitutive activity describes the ability of a GPCR to produce a biological response in the absence of a bound ligand [16, 17]. The degree of constitutive activity reflects the shift of the basal equilibrium from the inactive to the active state of a GPCR. Inverse agonists stabilize the inactive receptor conformation and are therefore capable of reducing or blocking constitutive activity. Consequently, constitutive activity of a GPCR is a prerequisite to determine inverse agonism and vice versa [18].

In contrast to the rodent orthologs mH_4_R and rH_4_R, high constitutive activity is characteristic of the hH_4_R [8, 9, 19, 20]. H_4_R species orthologs are well suited for exploring the molecular basis of this phenomenon, because there are not too many differences between the sequences in ECL2. Site-directed mutagenesis within the ECL2 of the hH_4_R compared to the mH_4_R revealed that the hH_4_R-F169V mutant is similar to the mH_4_R in terms of ligand affinities and potencies, suggesting that F169 is a key amino acid for differential interactions of certain agonists with the human and mouse H_4_R orthologs [21]. The assumption that F169 also contributes to constitutive activity was confirmed by investigations on the mutants hH_4_R-F169V and F169V+S179M [9]. F169 alone or in concert with S179 (TM5, ligand binding site) plays a major role in stabilizing a ligand-free active state of the hH_4_R. The constitutive activity of the hH_4_R-F169V mutant was significantly reduced compared to the wild-type hH_4_R. In particular, the inverse agonistic effect of thioperamide decreased.

F169 is part of the FF-motif, which is located on top of the ligand binding pocket (Fig. 1) and conserved in a number of class A GPCRs, e.g., the hβ_2_AR, hH_3_R, monkey H_4_R, canine H_4_R and the hM_2_R. Instead of the FF-motif, other GPCRs, such as the hβ_1_AR, hM_1_R, hM_3_R, hM_4_R, and the hM_5_R, as well as several H_4_R species orthologs, e.g., pig H_4_R, guinea pig H_4_R, mouse H_4_R and rat H_4_R, contain only one phenylalanine, which is located in a position corresponding to that of F168 in the hH_4_R. In these cases, in the adjacent position a non-aromatic hydrophobic amino acid such as valine or leucine is present instead of phenylalanine.

**Figure 2:**
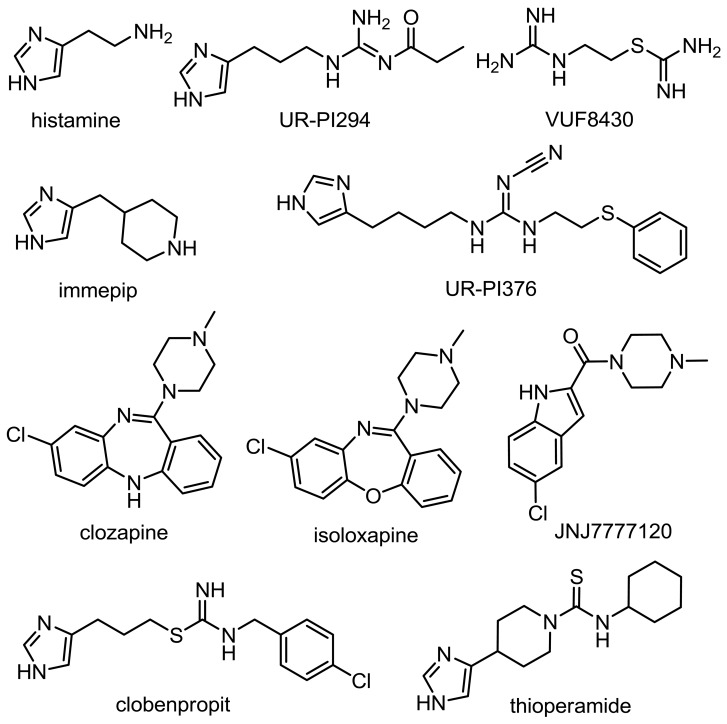
Structures of the investigated H_4_R ligands.

Crystal structures provide information on the position and the conformation of the FF-motif. The side chain of the first phenylalanine (in case of the hM_2_R also of the second one [22]) points into the ligand binding pocket. In the hβ_2_AR and in the hH_1_R, the second phenylalanine (and a tyrosine in case of hH_1_R) is oriented in the opposite direction [23, 24]. Our recent results on the contribution of F169 to the constitutive activity of the hH_4_R suggested that F168 plays a significant role as well. In order to investigate the influence of F168 on both receptor activation and ligand binding [9], we generated and characterized the hH_4_R-F168A mutant in comparison to the wild-type and the recently described hH_4_R-F169V mutant. The mutant receptors were expressed in Sf9 insect cells, and membrane preparations were used for saturation binding with [^3^H]histamine and functional studies were performed with inverse agonists, neutral antagonists and agonists in the [^35^S]GTPγS assay (Fig. 2).

## Materials and Methods

### Materials

The pcDNA3.1 vector containing the hH_4_R sequence was from the cDNA Resource Center at the University of Missouri-Rolla (Rolla, MO, USA). The pVL1392-SF-H_4_R-His_6_ plasmid was constructed as described previously [19, 25]. Baculovirus encoding Gα_i2_ was kindly provided by Dr. A. G. Gilman (Department of Pharmacology, University of Southwestern Medical Center, Dallas, TX USA). Recombinant baculovirus encoding the Gβ_1_γ_2_ subunits was a kind gift of Dr. P. Gierschik (Department of Pharmacology and Toxicology, University of Ulm, Ulm, Germany). *Pfu* Ultra II DNA polymerase was from Agilent (Böblingen, Germany). The DNA primers for polymerase chain reaction (PCR) were from MWG-Biotech (Ebersberg, Germany). Restriction enzymes were from New England Biolabs (Ipswich, MA USA). Gradient gels (8–16%, 12 well nUView gels) as well as the peqGOLD protein marker I, used for Coomassie brilliant blue R staining, were from Peqlab (Erlangen, Germany). UR-PI294 and UR-PI376 were synthesized as described [26, 27]. Thioperamide, JNJ7777120 and VUF8430 were synthesized according to Lange et al. [28], Jablonowski et al. [29], and Lim et al. [30]. Isoloxapine [31, 32] was synthesized and provided by Dr. S. Gobleder (Institute of Pharmacy, University of Regensburg, Regensburg, Germany). All other H_4_R ligands were from Tocris (Avonmouth, Bristol, UK). For chemical structures of the investigated compounds cf. Fig. 2. UR-PI376 (10 mM) was dissolved in 50% (*v/v*) dimethyl sulfoxide (DMSO) and dilutions were prepared in 20% (*v/v*) DMSO in order to attain a final DMSO concentration of 2% (*v/v*) in each well. Stock solutions (10 mM) of clozapine or isoloxapine were prepared in Millipore water containing 3 and 2 mol equivalents of HCl, respectively. All other stock solutions were prepared with Millipore water. [^35^S]GTPγS (1000 Ci/mmol) and [^3^H]histamine (25 Ci/mmol) were from Hartmann Analytic (Braunschweig, Germany). All other reagents were from standard suppliers and of the highest purity available.

### Methods

**Site-directed mutagenesis of the hH_4_R**. The preparation of the hH_4_R-F168A cDNA was essentially performed as described for the hH_4_R-F169V mutant [9]. To introduce the F168A mutation into the pVL1392-SF-hH_4_R-His_6_ expression vector a site-directed mutagenesis PCR was performed using the following primers 5’-GGT AGT GAA TGT GAA CCT GGA **GCC** TTT TCG GAA TGG TAC ATC C-3’ and 5’-G GAT GTA CCA TTC CGA AAA **GGC** TCC AGG TTC ACA TTC ACT ACC-3’.

**Cell culture, generation of recombinant baculoviruses and membrane preparation**. Cell culture and generation of high-titer recombinant baculovirus stocks [25] as well as the co-infection of Sf9 cells with high-titer baculovirus stocks encoding Gα_i2_, Gβ_1_γ_2_ and the respective H_4_R [8] were performed as described recently [9]. Membrane preparations were performed according to Gether et al. [33] in the presence of 0.2 mM phenylmethylsulfonyl fluoride, 1 mM ethylenediaminetetraacetic acid (EDTA), 10 μg/mL leupeptin and 10 μg/mL benzamidine as protease inhibitors. Prepared membranes were resuspended in binding buffer (75 mM Tris/HCl, 12.5 mM MgCl_2_,1 mM EDTA, pH 7.4) and stored at −80°C in 0.5 or 1.0 mL aliquots.

**SDS-PAGE and Coomassie staining**. Prior to incubation at 30°C for 15 min, the respective membrane preparation (15 µg protein) as well as a negative control (Sf9 cells transfected with pVL1392 devoid of an insert) were loaded onto the gel as well as 5 µL of the protein marker I [9]. A 2x sample buffer without urea was used for sample preparation. The gels were stained in a solution of 0.1% Coomassie brilliant blue G250 in 50% methanol and 10% acetic acid and subsequently destained with a solution containing 13% methanol and 7% acetic acid.

**[^3^H]histamine saturation binding experiments**. The experiments were performed in 96-well plates [9]. Each well contained 43–133 µg of protein in a total volume of 100 µL. For saturation binding, membranes were incubated in binding buffer containing [^3^H]histamine (1–200 nM) and 0.2% (*w/v*) BSA at room temperature under shaking at 200 rpm for 60 min. Non-specific binding was determined in the presence of 10 µM unlabeled histamine. Filtration through glass microfiber filters (Whatman GF/C), pretreated with polyethylenimine 0.3% (*w/v*), using a Brandel 96 sample harvester (Brandel, Unterföhring, Germany), was performed to separate unbound from membrane-associated [^3^H]histamine. After three washing steps with binding buffer, filter pieces were punched out, transferred into 96-well sample plates 1450–401 (Perkin Elmer, Rodgau, Germany), and 200 µL of scintillation cocktail (Rotiscint Eco plus, Roth, Karlsruhe, Germany) per well were added before incubation in the dark under shaking at 200 rpm. Radioactivity was measured with a Micro Beta2 1450 scintillation counter (Perkin Elmer, Rodgau, Germany).

**[^35^S]GTPγS binding assay**. Membranes were thawed, centrifuged for 10 min at 4°C and 13,000 g and carefully resuspended in binding buffer [9]. Experiments were performed in 96-well plates in a total volume of 100 µL per well. Each well contained 7–19 µg of protein (7–10 µg for hH_4_R, 10–14 µg for hH_4_R-F169V and 10–19 µg for hH_4_R-F168A), 1 µM GDP, 100 mM NaCl, 0.05% (*w/v*) bovine serum albumine (BSA), 20 nCi of [^35^S]GTPγS (0.2 nM) and ligand at concentrations as indicated in the results section. Antagonism was determined in the presence of histamine (10-fold EC_50_ at the respective receptor). Nonspecific binding was determined in the presence of 10 µM unlabeled GTPγS. After incubation under shaking at 200 rpm at room temperature for 2 h, bound [^35^S]GTPγS was separated from free [^35^S]GTPγS by filtration through glass microfibre filters using a 96-well Brandel harvester. The filters were washed three to four times with binding buffer (4°C), dried over night and impregnated with meltable scintillation wax prior to counting with a Micro Beta2 1450 scintillation counter.

Protein concentrations of all membrane preparations were determined with the Bio-Rad DC protein assay kit (München, Germany) in one experiment. Because UR-PI376 had to be dissolved in 20% DMSO, the water control as well as the full agonist histamine (α = 1.0), to which all other ligands were referenced, were also dissolved in 20% DMSO in case of this ligand. Concentration-response curves were constructed by fitting the data according to the four parameter logistic fit (variable slope), and analyzed with the Prism 5.01 software (GraphPad, San Diego, CA USA). *K_b_* values were calculated according to the Cheng-Prusoff equation [34]. All values are given as mean ± SEM of at least three independent experiments performed in triplicate. Significances were calculated using one-way analysis of variance (ANOVA), followed by Bonferroni’s multiple comparison test.

## Results

### Receptor expression

**Figure 3:**
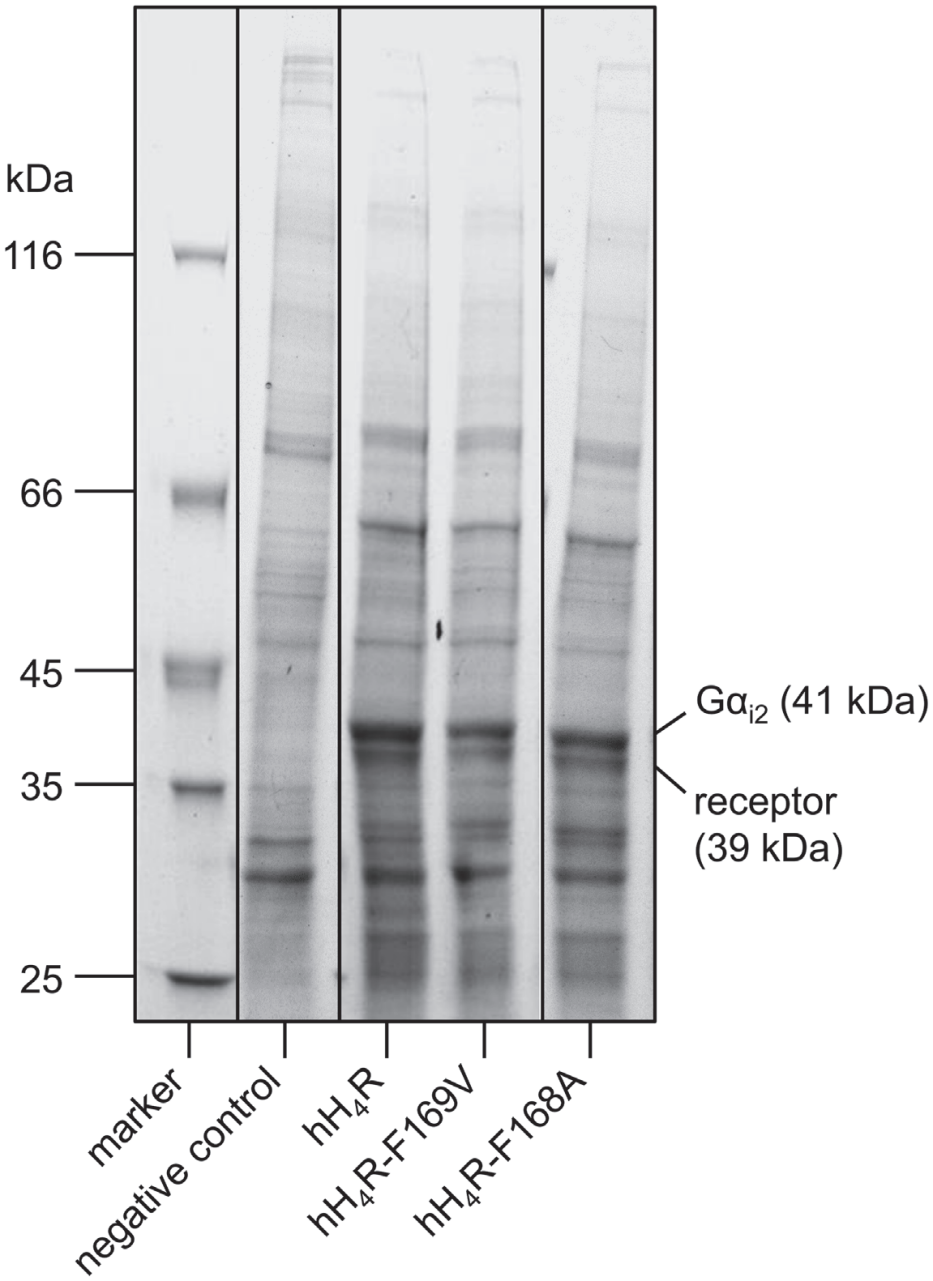
Coomassie stained SDS gels. Membrane proteins of Sf9 insect cells, coexpressing the respective receptor as indicated and Gα_i2_ as well as Gβ_1_γ_2_ were separated on 8–16% polyacrylamide gradient gels. All samples were analyzed on the same gel. In the interest of clarity, the membranes prepared from Sf9 cells transfected with pVL1392 devoid of an insert (negative control) were placed next to the molecular weight standard.

Human histamine H_4_ receptor wild-type as well as mutants (hH_4_R-F169V and hH_4_R-F168A) were expressed in Sf9 insect cells together with G-protein subunits Gα_i2_ and Gβ_1_γ_2_ [9, 35]. As previously shown by SDS PAGE and western blots [9], the wild-type or mutated H_4_ receptors migrated with an apparent molecular weight of 39 kDa and the Gα_i2_ protein with an apparent molecular weight of 41 kDa. The hH_4_R wild-type and both mutant receptors, hH_4_R-F169V and hH_4_R-F168A, respectively as well as the Gα_i2_ protein were expressed at comparably high levels as becomes obvious from Coomassie stained SDS gels (Fig. 3). However, specific binding of [^3^H]histamine to the hH_4_R-F168A mutant was too low to determine the K_d_ value (highest concentration of radioligand used: 200 nM). By contrast, the wild-type hH_4_R as well as the hH_4_R-F169V mutant revealed high specific binding as described previously (cf. [9], saturation binding curves are depicted in Figures S3A and B in the Supporting Information of the respective publication).

### Functional analysis of wild-type and mutant H_4_ receptors

**Figure 4:**
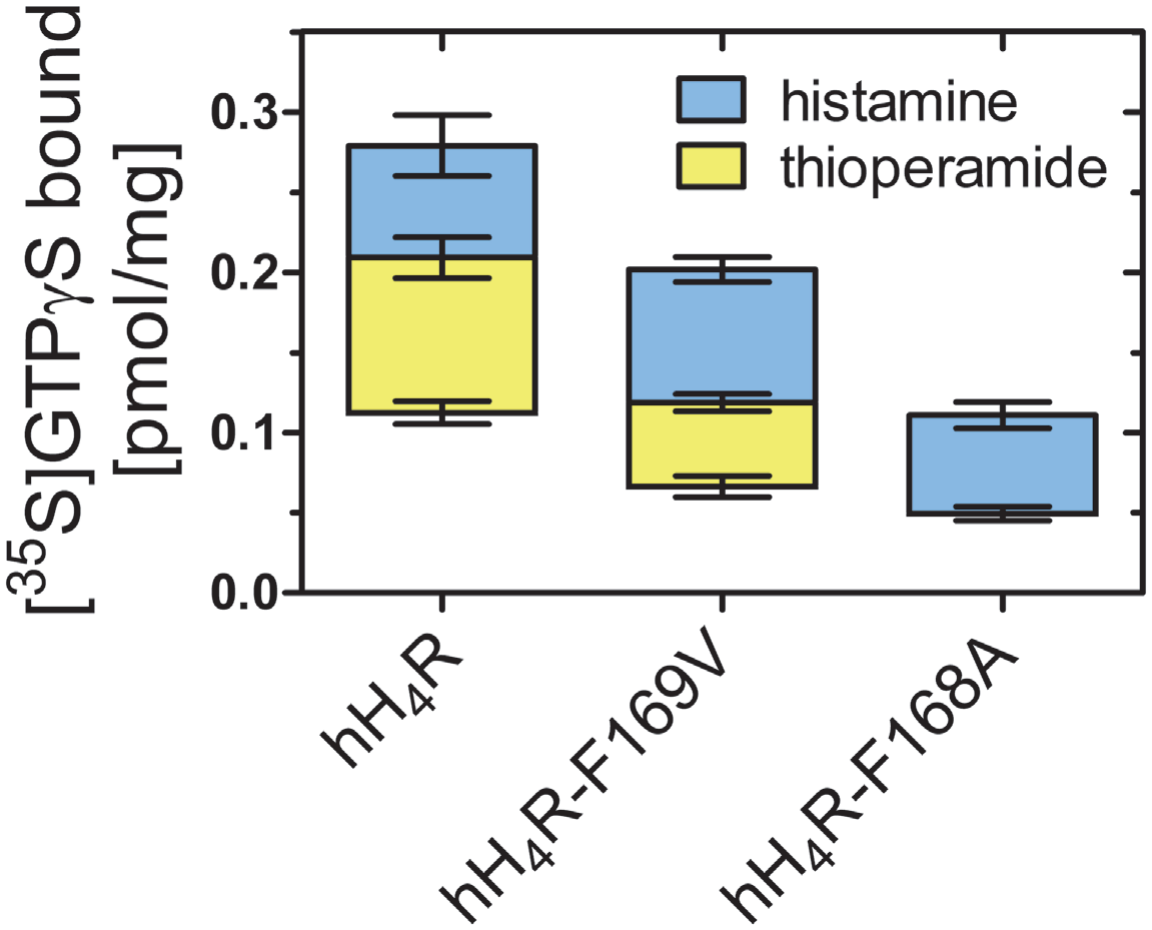
Maximal agonistic effects of histamine (light blue) and maximal inverse agonistic effects of thioperamide (yellow) in the [^35^S]GTPγS-assay. Data represent [^35^S]GTPγS [pmol/mg protein] specifically bound to wild-type and mutated H_4_Rs. The line separating light blue and yellow bar represents [^35^S]GTPγS binding in the absence of ligand.

Functional data—intrinsic activities (α), potencies (pEC_50_) and antagonist activities (pK_b_)—were determined in the [^35^S]GTPγS assay using standard agonists as well as inverse agonists and neutral antagonists (Fig. 2 and Table 1). For comparison, data from the hH_4_R-F169V mutant [9] are included in Table 1. Upon maximal stimulation with histamine, the amounts of bound [^35^S]GTPγS were significantly different, decreasing in the order hH_4_R wild-type > hH_4_R-F169V > hH_4_R-F168A (Fig. 4). The effect of the inverse agonist thioperamide reflects constitutive activity of wild-type and mutant receptors. The response to thioperamide decreased in the order hH_4_R > hH_4_R-F169V > hH_4_R-F168A (Fig. 4), i. e., constitutive activity was highest at the hH_4_R wild-type, significantly smaller at the hH_4_R-F169V mutant [9] and absent at the hH_4_R-F168A mutant, where thioperamide acted as a neutral antagonist.

**Table 1:** [^35^S]GTPγS binding on hH_4_R wild-type, hH_4_R-F169V and hH_4_R-F168A mutant.

**Ligand**	**Parameter**	**hH_4_R**	**hH_4_R-F169V**	**hH_4_R-F168A**
histamine	α	1	1	1
	pEC_50_	8.13 ± 0.06	7.72 ± 0.07 ^●●^	5.98 ± 0.06 ^●●●^
UR-PI294	α	1.02 ± 0.03	1.00 ± 0.07	0.91 ± 0.06
	pEC_50_	8.35 ± 0.04	8.00 ± 0.11	6.78 ± 0.11 ^●●●^
thioperamide	α	−1.39 ± 0.08	−0.63 ± 0.06 ^●●●^	0 ^●●●^
	pEC_50_	6.58 ± 0.06	6.52 ± 0.05	n.a.
	pK_b_	6.83 ± 0.05		7.97 ± 0.07 ^●●●^
JNJ7777120	α	−0.39 ± 0.03	0.43 ± 0.03 ^●●●^	0.20 ± 0.01 ^●●●^
	pEC_50_	7.10 ± 0.08	6.21 ± 0.12 ^●●^	6.40 ± 0.17
	pK_b_	7.60 ± 0.05		6.17 ± 0.19 ^●●^
VUF8430	α	0.84 ± 0.06	0.91 ± 0.06	0.69 ± 0.06
	pEC_50_	7.42 ± 0.12	7.61 ± 0.07	5.74 ± 0.03 ^●●●^
Immepip	α	0.81 ± 0.03	0.85 ± 0.05	0.81 ± 0.02
	pEC_50_	7.67 ± 0.05	7.73 ± 0.19	5.82 ± 0.11 ^●●●^
clozapine	α	0.67 ± 0.04	0.56 ± 0.03	0.40 ± 0.01 ^●●^
	pEC_50_	6.24 ± 0.10	5.68 ± 0.12 ^●^	5.38 ± 0.10 ^●●^
isoloxapine	α	0.81 ± 0.03	0.85 ± 0.09	0.83 ± 0.07
	pEC_50_	7.08 ± 0.13	6.36 ± 0.10 ^●●^	6.10 ± 0.05 ^●●●^
UR-PI376	α	1.11 ± 0.08	0.49 ± 0.02 ^●●●^	0.39 ± 0.05 ^●●●^
	pEC_50_	7.79 ± 0.08	6.25 ± 0.11 ^●●●^	6.30 ± 0.15 ^●●●^
clobenpropit	α	0.45 ± 0.04	0.27 ± 0.05 ^●^	0.14 ± 0.02 ^●●^
	pEC_50_	7.65 ± 0.11	7.63 ± 0.15	7.40 ± 0.13
	pK_b_			7.24 ± 0.06

pEC_50_-values ([^35^S]GTPγS agonist mode), pK_b_-values ([^35^S]GTPγS antagonist mode) and α (intrinsic activity, maximal effect relative to histamine = 1.0) are given as mean ± SEM of at least three independent experiments, performed in triplicate. Results of statistical tests (one-way ANOVA and Bonferroni-posthoc-tests): significant differences with respect to hH_4_R-

^●^ p < 0.05,

^●●^ p < 0.01,

^●●●^ p < 0.001.

In case of neutral antagonism (−0.25 ≤ α ≤ 0.25), pK_b_-values were considered for statistical analysis instead of pEC_50_-values. Maximal effect α = 0: neutral antagonism. Data for hH_4_R and hH_4_R-F169V cf. Wifling et al. [9].

**Figure 5:**
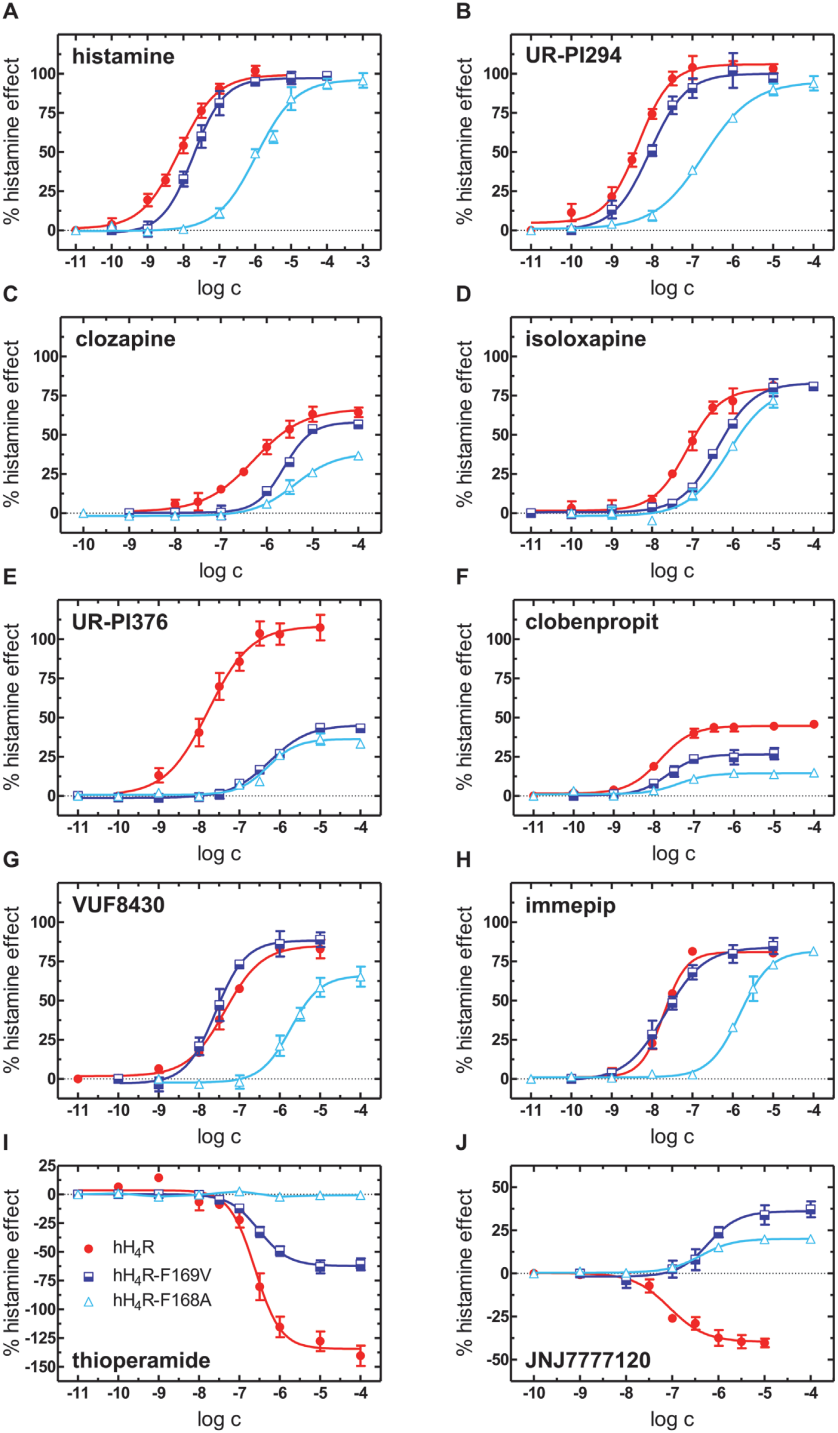
Concentration-response curves of ligands investigated in the [^35^S]GTPγS assay. All curves are normalized with respect to the maximal effect of histamine (100%) at the respective receptor.

The normalized concentration-response curves of histamine (maximal effect of histamine at the respective receptors, set to 100%) are depicted in Fig. 5A. The potency of histamine decreased from the hH_4_R via the hH_4_R-F169V to the hH_4_R-F168A mutant by more than two orders of magnitude (Fig. 5A and Table 1). The same holds for the full agonist UR-PI294 [27] with a decrease in potency by about 1.5 orders of magnitude from the hH_4_R to the hH_4_R-F168A mutant without significant changes of intrinsic activity (Fig. 5B).

The potency of clozapine and the structurally related isoloxapine decreased from the hH_4_R via the hH_4_R-F169V to the hH_4_R-F168A mutant with maximal shift of the curve by one order of magnitude (Fig. 5C, D). The intrinsic activity of clobenpropit, a partial agonist, and UR-PI376 [26], a full agonist at the hH_4_R significantly decreased at the two mutants (Fig. 5E, F). For clobenpropit, despite reduced maximal responses, no significant changes of the potency were observed. By contrast, the potency of UR-PI376 was by more than one order of magnitude lower at the mutants than at the wild-type.

Compared to the wild-type hH_4_R, the potencies and intrinsic activities of the partial agonists immepip and VUF8430 were not significantly affected by the hH_4_R-F169V mutation [9]. By contrast, at the hH_4_R-F168A mutant, the potencies decreased by about two orders of magnitude (Fig. 5G, H).

Inverse agonism of thioperamide was highest at the hH_4_R, significantly lower at the hH_4_R-F169V [9] and not detectable at the hH_4_R-F168A mutant (Fig. 5I). Instead, thioperamide behaved as a neutral antagonist with a pK_b_ value of 7.97. JNJ7777120 was a partial inverse agonist at the hH_4_R but, surprisingly, acted as a partial agonist at the hH_4_R-F169V and hH_4_R-F168A mutants (Fig. 5J).

## Discussion

### Potencies of ligands at mutated H_4_ receptors

With respect to potency at mutant H_4_ receptors, except thioperamide, the investigated ligands are divided in two groups. The first group, comprising JNJ7777120, clozapine, isoloxapine, UR-PI376 and clobenpropit, has similar potency at both the hH_4_R-F169V and the hH_4_R-F168A mutant. These ligands contain bulky aromatic groups. The phenyl and chlorophenyl moieties of clozapine and JNJ7777120, respectively, were suggested to occupy a hydrophobic pocket between TMs 3, 5, 6 and ECL2 [36, 37]. Most notably, MD simulations with JNJ7777120 indicated that the chloro substituent is surrounded by a relatively tight pocket formed by E163^ECL2^, F168^ECL2^, F169^ECL2^, L175^5.39^ and T323^6.55^ [38]. Mutations of these amino acids, especially, affect binding modes directed towards ECL2. Affinity of ligands may be reduced due to loss of direct contacts and/or by distortion of the pocket. The binding mode of clobenpropit is probably different, because of similar potency at the wild-type and both mutants.

**Figure 6:**
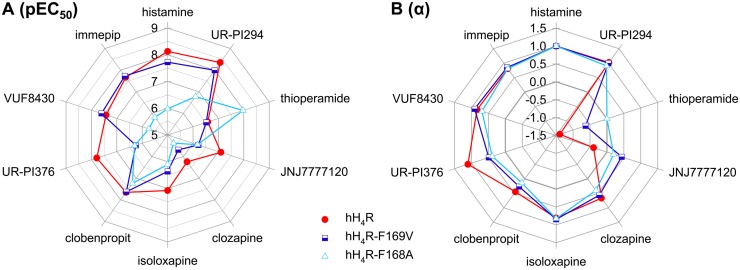
Radar plots of potencies and maximal effects at wild-type human H_4_R, hH_4_R-F169V and hH_4_R-F168A mutants. (A) pEC_50_ values (or pK_b_ in case of partial agonists/inverse agonists with −0.25 ≤ α ≤ 0.25). (B) maximal effects (α values, relative to histamine = 1).

The second group, histamine, UR-PI294, VUF8430 and immepip, comprises rather small ligands devoid of hydrophobic substituents. Characteristic of this group is a significant decrease in potency by about two orders of magnitude at the hH_4_R-F168A mutant compared to the wild-type hH_4_R (Fig. 6A). By contrast, there were only minor effects on potency at the hH_4_R-F169V mutant. Thus, F168 is probably involved in direct interactions with the ligands of this group.

The ligands of both groups are full or partial agonists, apart from JNJ7777120 at the wild-type hH_4_R. According to docking on hH_4_R homology models, agonists as well as several antagonists and inverse agonists probably bind between TMs 3, 5, 6 and 7 via key interactions with D94^3.32^, E182^5.46^ and Q347^7.42^ [36–38]. Thioperamide is an exception as it binds only to inactive hH_4_R state(s). By analogy, thioperamide is known to stabilize the inactive conformation of the closely related hH_3_R. Molecular dynamics simulations of an hH_3_R-thioperamide complex revealed a binding mode characterized by an extended conformation of the ligand, which is oriented parallel to the membrane plane, an interaction of the imidazolyl moiety with tyrosine in position 2.61, and the thiourea group positioned in the vicinity of F193, which corresponds to F169 in the hH_4_R [39]. It may be speculated that thioperamide binds to the hH_4_R in the same way, selectively contacting Y72^2.61^ and F344^7.39^, whereas interactions with E182^5.46^ and Q347^7.42^, proven essential in case of other H_4_R ligands, are precluded or only weak. Such a binding mode would prevent the constriction of the orthosteric binding site (inward movements of TMs 5, 6 and 7), characteristic of the conversion of the receptor to the active state [23]. Direct interactions of thioperamide with F168 or F169 cannot be deduced from the data in Table 1. The increase in pK_b_ at the hH_4_R-F168A mutant by one order of magnitude compared to the wild-type receptor is compatible with higher affinity of thioperamide to inactive than to active state(s), represented by the mutant devoid of constitutive activity and the highly constitutively active wild-type H_4_R.

### Intrinsic activities of ligands and constitutive activity of receptors

The hH_4_R agonists histamine, UR-PI294, isoloxapine, VUF8430 and immepip did not show significantly reduced intrinsic activities at both hH_4_R mutants compared to the wild-type, whereas the maximal effects of clozapine, clobenpropit and UR-PI376 were diminished (Fig. 6B). In case of inverse agonists, the reduced constitutive activity of the mutants is reflected by lower maximal (inverse) responses. The partial inverse hH_4_R agonist JNJ7777120 was a partial agonist at the mutant receptors. Thioperamide was a partial inverse agonist at hH_4_R-F169V, the mutant with reduced constitutive activity, and a neutral antagonist at the hH_4_R-F168A mutant, which is devoid of constitutive activity. The results support the hypothesis that both F168 and F169 play a role in stabilizing an active state of the wild-type hH_4_R.

Constitutive activity [16] reflects a ligand-independent interconversion between inactive and active receptor conformations. Interactions at the intracellular face involving the DRY motif have been proven crucial for basal and agonist-induced receptor activation and signaling [35, 40]. In case of the hH_4_R, which is devoid of the ionic lock, we demonstrated that interactions close to the ligand binding pocket and ECL2 account for the high constitutive activity [9]. The mutation of F169 alone and, even more pronounced, the mutation of both F169 (ECL2) and S179^5.43^ (numbering according to the Ballesteros nomenclature [41]) into the corresponding amino acids of the mouse and rat H_4_R orthologs (F169V, S179M, S179A) resulted in a highly significant reduction of the constitutive activity [9].

**Figure 7:**
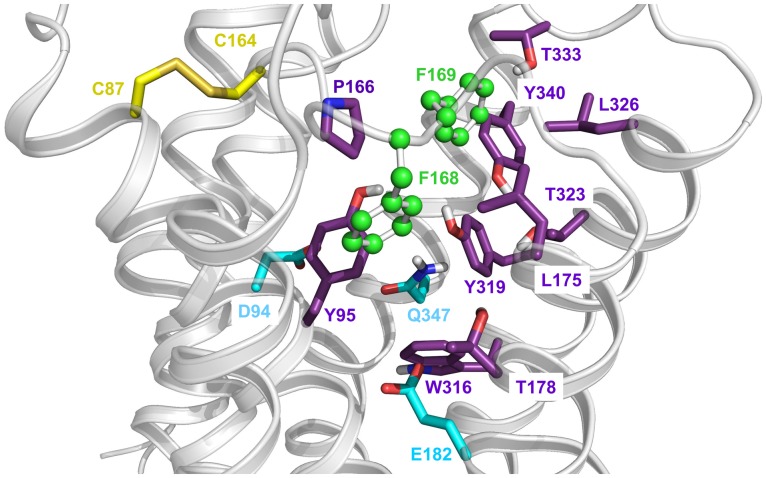
Binding pocket of the hH_4_R, homology model [9] based on the inactive state crystal structure of the hH_1_R [24]. Nitrogens are colored in blue, oxygens in red and sulfurs in yellow. The carbons are differently colored: the two cysteines forming the disulfide bond in yellow, the amino acids representing the hydrophobic cluster in magenta, important amino acids for ligand binding in cyan and the two adjacent phenylalanines forming the FF-motif in green.

The hH_4_R model in Fig. 7 suggests mutual effects of both phenylalanines, F168 and F169 (the FF motif), on the conformation of ECL2 [21]. Our present results with the hH_4_R-F168A mutant support this idea. Compared to hH_4_R-F169V, which has still a low constitutive activity, hH_4_R-F168A is completely devoid of constitutive activity. Accordingly, the single mutation of either F169 into V and, especially, F168 into A weakens interactions within ECL2 and the surrounding hydrophobic pocket consisting of amino acids as Y95^3.33^, P166^ECL2^, L175^5.39^, T178^5.42^, T323^6.55^, L326^6.58^, T333^ECL3^, and Y340^7.35^ (Fig. 7). Therefore, replacement of F168 or F169 probably causes major conformational changes, which destabilize active and stabilize inactive receptor states.

## Conclusions

The present study demonstrates a highly significant influence of the hH_4_R-F168A mutant on ligand binding as well as on constitutive activity, even surpassing the consequences of hH_4_R-F169V mutation, revealing a key role of the FF motif for both, ligand-receptor interaction and interconversion between inactive and active conformation of the wild-type hH_4_R. The results may also be of relevance for other class A GPCRs comprising the FF motif, such as the β_2_AR, the H_3_R and the M_2_R.
