# Correction: The Extracellular Loop 2 (ECL2) of the Human Histamine H4 Receptor Substantially Contributes to Ligand Binding and Constitutive Activity

**DOI:** 10.1371/journal.pone.0122162

**Published:** 2015-03-30

**Authors:** The PLOS ONE Staff

The PDF version of Fig. 2 is incorrect. The online version of Fig. 2 is correct, and the authors have also provided a corrected version here.

**Fig 2:**
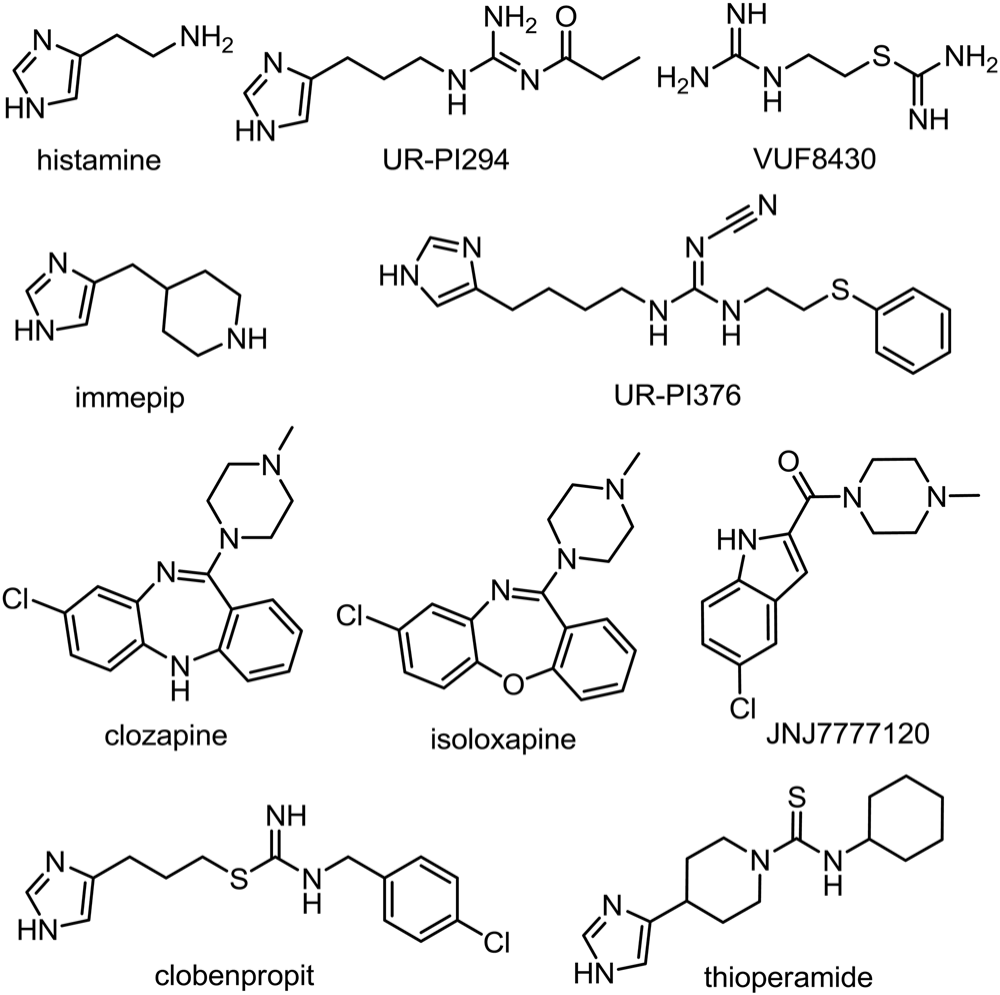
Structures of the investigated H4R ligands.

## References

[1] Wifling D, Bernhardt G, Dove S, Buschauer A (2015) The Extracellular Loop 2 (ECL2) of the Human Histamine H4 Receptor Substantially Contributes to Ligand Binding and Constitutive Activity. PLoS ONE 10(1): e0117185 doi: 10.1371/journal.pone.0122162 25629160 10.1371/journal.pone.0117185 PMC4309601

